# Effect of response quality and line of treatment with rituximab on overall and disease-free survival of patients with B-cell lymphoma

**DOI:** 10.2478/v10019-010-0044-6

**Published:** 2010-10-14

**Authors:** Mateja Horvat, Barbara Jezersek Novakovic

**Affiliations:** Department of Medical Oncology, Institute of Oncology Ljubljana, Slovenia

**Keywords:** B-cell lymphoma, rituximab, response quality, the line of treatment, overall survival, disease-free survival

## Abstract

**Background:**

The introduction of rituximab into the treatment of patients with non-Hodgkin’s lymphomas has improved the overall response rate, as well as the response duration and the overall survival of patients with B-cell lymphomas. But only a few studies have addressed the question whether the better response (complete response) and the early introduction of rituximab into the treatment translate into the better survival. The aim of this retrospective study was to assess the potential relationship between either the quality of the response or the line of the rituximab treatment and the overall survival (OS) as well as the disease-free survival (DFS) of patients with B-cell lymphomas.

**Patients and methods.:**

In the study, we analysed treatment outcomes in patients with different histological types of B-cell lymphomas who were treated at the Institute of Oncology between 2003 and 2007 with rituximab and chemotherapy. We included only patients who had the level of CD20 expression assessed prior to the introduction of the treatment with quantitative flow-cytometric measurements. The OS and DFS were evaluated by Kaplan-Meier survival curves.

**Results:**

One hundred and fourteen patients were enrolled in the study. Patients who achieved a complete response after the rituximab containing treatment had a significantly longer OS than those reaching a partial response (hazard ratio [HR], 0.34; 95% CI, 0.05 to 0.91, P = 0.0375) and than patients with stable (hazard ratio [HR], 0.11; 95% CI, 0.0002 to 0.033, P < 0.0001) or progressive disease (hazard ratio [HR], 0.09; 95% CI, 0.003 to 0.03, P < 0.0001). Patients who achieved a complete response (CR; n = 70; 61.4%) had also a significantly longer DFS (hazard ratio [HR], 0.26; 95% CI, 0.021 to 0.538, P = 0.0068) than those reaching only a partial response (PR; n = 17; 14.9%). Patients treated with rituximab as the first-line treatment (n = 50; 43.9%) had a significantly longer OS than those treated with rituximab for the first (hazard ratio [HR], 0.27; 95% CI, 0.106 to 0.645, P = 0.0036) or second relapse (hazard ratio [HR], 0.22; 95% CI, 0.078 to 0.5, P = 0.0006). Also the DFS of patients treated with rituximab as the first-line treatment (n = 46; 52.9%) was significantly longer (hazard ratio [HR], 0.32; 95% CI, 0.088 to 0.9, P = 0.0325) than in patients treated with rituximab for their first relapse (n = 25; 28.7%).

**Conclusions:**

These data indicate that a better response to rituximab therapy presumably translates into an improved OS and DFS for patients with B-cell lymphomas. The early introduction of rituximab into the treatment (i.e. first-line treatment) might improve OS. Therefore, the response adapted first-line therapy with rituximab should be considered when the treatment decision is taken in B-cell lymphoma patients.

## Introduction

B-cell lymphomas are a group of diseases characterized by the proliferation of lymphoid tissue and occasionally by the presence of abnormal lymphoid elements in the peripheral blood.^1^ The incidence of these malignancies has been increasing over the past several decades by approximately 3% per year.^2^ Over the last two decades, there has been a significant increase in management options of these patients, consisting of the observation in case of indolent lymphomas, various chemotherapies (alkylating agent-based, fludarabine-based, antracycline-based), hematopoietic stem-cell transplantation and biologic therapies among which also the therapy that targets the CD20 antigen, such as rituximab.

The introduction of rituximab into the treatment of patients with non-Hodgkin lymphoma has improved the overall response rate, as well as the response duration and the overall survival of patients with B-cell lymphomas.^3^,^4^ Although there has been some evidence that in the treatment of lymphoma patients the better response is associated with the prolonged disease-free survival, the correlation between the quality of response and the survival still remains unknown.^4^–^9^ Recently published studies have indicated that a better response to first-line treatment translates into an improved survival for patients with follicular lymphoma and also with other malignomas.^10^,^11^ With our retrospective study we, therefore, wanted to assess the potential correlation between either the quality of response or the line of the rituximab treatment with both the overall survival (OS) and the disease-free survival (DFS) of patients with B-cell lymphomas.

## Patients and methods

### Patient population

Patients with different histological types of B-cell lymphomas treated with the rituximab containing therapy between 2003 and 2007 at the Institute of Oncology Ljubljana were included in our retrospective study. Because this study was a part of a more extensive research on the correlation between the CD20 expression and treatment outcome just patients who had the level of CD20 expression assessed prior to the introduction of the treatment with quantitative flow-cytometric measurements were selected. These patients represented approximately 33.5% of all patients treated routinely with rituximab in that period.

Patients received treatments according to the then protocol at our Institute. Aggressive lymphomas were predominately treated with the rituximab containing therapy in the first-line and just relapsing patients who had not previously received rituximab were treated in the second or consecutive lines. On the other hand, most patients with indolent lymphomas were in line with the recommendations not treated with the rituximab containing therapy until relapse. Rituximab – chemotherapy combinations were selected according to the histological type of lymphoma, extent of the disease and previous treatments. Most of the patients received R-CHOP (75.4%). Patients with a more limited disease were planned to receive 6 and patients with extensive disease up to 8 chemo-immunotherapy cycles.

### Response to treatment

The treatment response data were retrospectively noted from patients’ records for the specified B-cell lymphoma patients. The quality of the response was assessed by Cheson’s criteria.^12^

In the next step, the OS data for all included B-cell lymphoma patients together with the DFS data for the B-cell lymphoma patients who achieved the complete (CR) or the partial response (PR) after the treatment were obtained.

The OS was assessed by Kaplan-Meier survival curves from the beginning of the treatment for all patients. The response duration expressed as the DFS was evaluated by Kaplan-Meier survival curves from the end of the treatment for the patients attaining CR or PR after the rituximab containing treatment.

### Statistical analysis

The difference between the survival curves was assessed by a log-rank test and the difference between the overall response rates by a hi-square test.

## Results

### Characteristics of the study participants

One hundred and fourteen patients with different histological types of B-cell lymphomas who were treated with rituximab and chemotherapy between 2003 and 2007 and had the level of CD20 expression assessed prior to the introduction of the treatment with quantitative flow-cytometric measurements were included. There were 64 males and 50 females with the median age of 58.5 years (range 19 to 82 years). The majority of patients had the diffuse large B-cell lymphoma (DLBCL; 42 patients; 36.8%) and follicular lymphoma (FL; 30 patients; 26.3%). Patients’ characteristics together with the distribution of patients according to the lines of the treatment are given in Table 1.

In the majority of patients, rituximab was given as the first-line treatment (50 patients; 43.9%), while 33 patients (28.9%) received it as the second-line treatment, and 31 patients (27.2%) as the third or subsequent line of the treatment. There was, however, some difference between aggressive and indolent lymphomas – namely 78.6% of patients with aggressive lymphomas received rituximab as the first-line treatment, 19% as the second-line treatment and 2.4% as the third/subsequent line treatment while just 23.6% of patients with indolent or unclassified lymphomas were treated with rituximab as the first-line, 34.7% as the second-line and 41.7% as the third/subsequent line of the treatment (Table 1).

All patients received at least 3 and up to 8 cycles of the treatment. No patients were excluded due to serious side effects. The follow-up of side effects was by all means not the objective of this study, yet we have to mention that in the routine setting serious side effects of the rituximab treatment have been during a longer period of time observed in less than 1% of patients.

### The effect of the response quality on OS and DFS

The overall response rate (ORR) of the treatment with rituximab and chemotherapy regardless of the histological type of lymphoma or of the line of treatment was 76.3% (61.4% CR; 14.9% PR). The distribution of patients receiving rituximab according to the response quality is given in Table 2.

Table 3 additionally gives the distribution of patients receiving rituximab according to the response quality and the type of lymphoma, yet the data are given just for those lymphoma type groups that comprise more than 10 patients.

**FIGURE 1.**
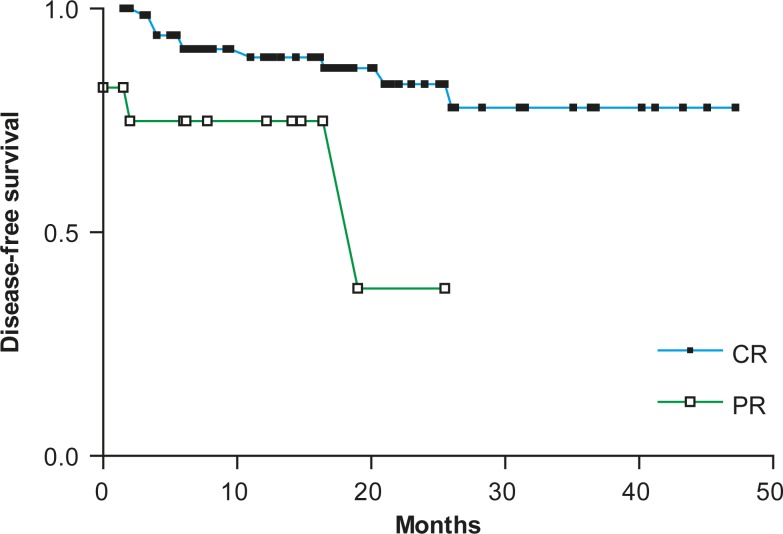
Disease-free survival after treatment with rituximab and chemotherapy according to the response quality. *CR* - complete response, *PR* - partial response

In continuation, the DFS was evaluated from the end of the treatment for patients achieving CR and PR to the treatment with rituximab and chemotherapy (87 patients; 76.3%) and is according to the response quality presented in Figure 1.

The B-cell lymphoma patients treated with rituximab who achieved a complete response (CR; n = 70; 61.4%) had a significantly longer DFS (hazard ratio [HR], 0.26; 95% CI, 0.021 to 0.538, P = 0.0068) than those reaching just a partial response (PR; n = 17; 14.9%). In patients who achieved CR, the median response duration has not been reached yet, while for the patients who achieved PR, it was 19 months.

**FIGURE 2.**
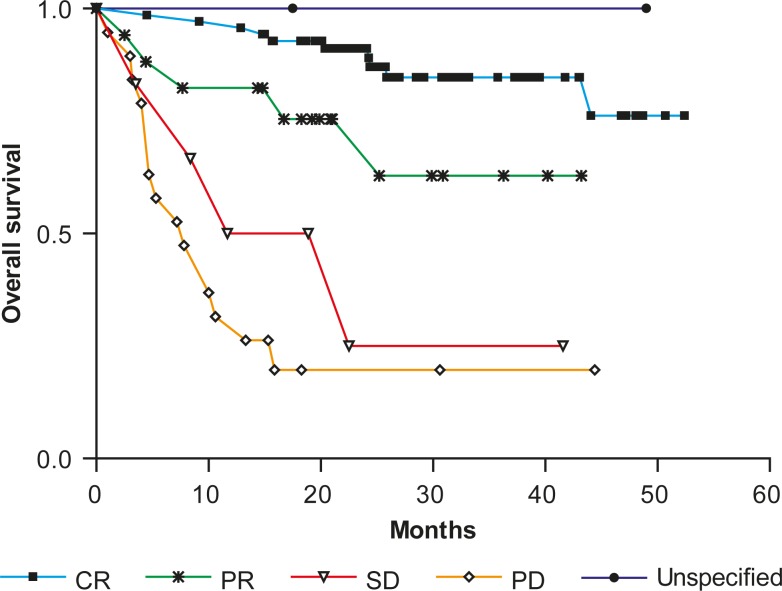
Overall survival after treatment with rituximab and chemotherapy according to the response quality. *CR* - complete response, *PR* - partial response, *SD* - stable disease, *PD* - progressive disease, *ORR* - overall response rate, *Unspecified* – response not specified

The OS was assessed from the beginning of the treatment for all 114 patients treated with rituximab and chemotherapy. Figure 2 presents the OS after the treatment with rituximab and chemotherapy according to the response quality.

The B-cell lymphoma patients who achieved a complete response after the rituximab therapy had a significantly longer OS than those reaching a partial response (hazard ratio [HR], 0.34; 95% CI, 0.05 to 0.91, P = 0.0375), and than patients with stable (hazard ratio [HR], 0.11; 95% CI, 0.0002 to 0.033, P <0.0001) or progressive disease (hazard ratio [HR], 0.09; 95% CI, 0.003 to 0.03, P <0.0001). Patients who achieved a partial response had a significantly longer OS than those with progressive disease (hazard ratio [HR], 0.24; 95% CI, 0.095 to 0.589, P = 0.002). In patients with stable disease after the rituximab therapy, the median OS was 17.1 months, while for the patients with progressive disease, it was only 7.8 months. The median OS has not been reached for patients with CR, PR and unspecified response.

### The effect of the line of the treatment with rituximab on OS and DFS

The distribution of patients receiving rituximab according to the response quality and the line of the treatment is given in Table 4.

**FIGURE 3.**
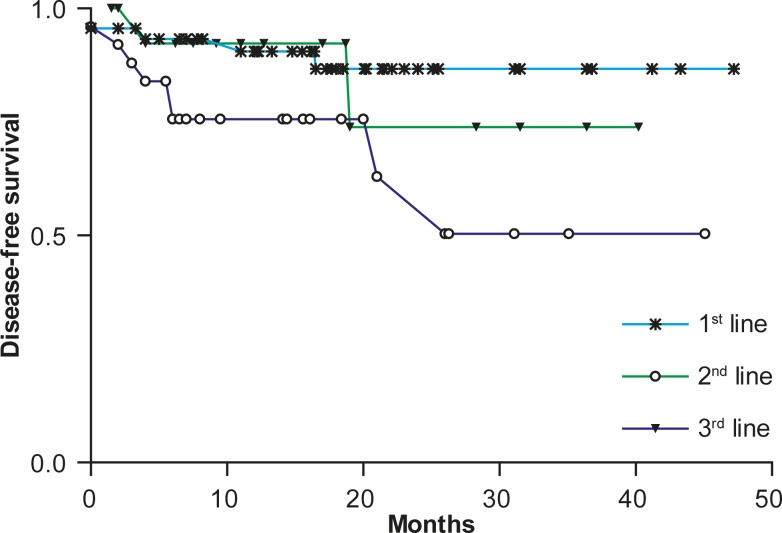
Disease-free survival after treatment with rituximab and chemotherapy according to the lines of treatment.

The duration of the response expressed as the DFS for those 87 patients (76.3%) achieving CR and PR to the treatment with rituximab and chemotherapy is according to lines of the treatment shown in Figure 3.

The difference in response duration was statistically insignificant either between the patients treated with rituximab as the third or subsequent line (n = 16; 16%) and first-line treated lymphoma patients (n = 46; 52.9%) or between the patients treated with rituximab as the third or subsequent line (n = 16; 16%) and second-line treated lymphoma patients (n = 25; 28.7%). However, first-line treated lymphoma patients (n = 46; 52.9%) had a significantly longer DFS (hazard ratio [HR], 0.32; 95% CI, 0.088 to 0.9, P = 0.0325) than patients treated with rituximab for the first relapse (n = 25; 28.7%). The median DFS has not been reached for any patient group treated with rituximab.

**FIGURE 4.**
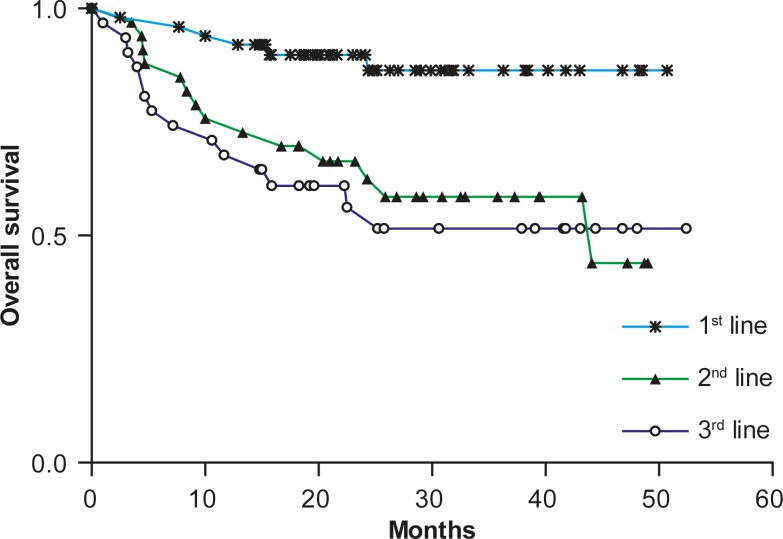
Overall survival after treatment with rituximab and chemotherapy according to the lines of treatment.

The OS from the beginning of the treatment for all 114 patients treated with rituximab and chemotherapy according to the lines of the treatment is presented in Figure 4.

First-line treated lymphoma patients (n = 50; 43.9%) had a significantly longer OS than those treated with rituximab for the first (hazard ratio [HR], 0.27; 95% CI, 0.106 to 0.645, P = 0.0036) or the second relapse (hazard ratio [HR], 0.22; 95% CI, 0.078 to 0.5, P = 0.0006). The median OS in patients treated with rituximab as the second-line treatment was 44.1 months. The median OS has not been reached for patients treated with rituximab as the first or third line of the treatment.

## Discussion

We analyzed the potential influence of response quality and the line of the treatment on OS and DFS of patients with different histological types of B-cell lymphomas. Results of our study provide some evidence that response quality parallels with the survival since patients who achieved a complete response had a significantly longer OS than those reaching a partial response or those experiencing stable or progressive disease. Yet, we must be aware that our study included both patients with indolent and patients with aggressive subtypes of lymphomas. As the two groups of B-cell lymphomas differ in their natural course, aggressiveness and above all in their susceptibility to chemo-immunotherapy, this result may have been partially influenced by the rather large proportion (36.8%) of aggressive lymphoma patients in our study. Aggressive lymphomas differ from the indolent ones as they can be to a certain extent cured with conventional chemotherapy or chemo-immunotherapy. The goal of the treatment of aggressive lymphomas is, therefore, the achievement of as many as possible complete responses in the frontline treatment since only those patients are expected to be cured.

On the other hand, up till now quite a few studies in indolent follicular lymphomas evidenced that a better response is associated with prolonged DFS yet the correlation between the quality of response and OS has not been unequivocally confirmed.^4^–^8^ This might be at least partially on account of a rather short follow-up in these studies since an improved long-term survival for follicular lymphoma patients who reached a CR in the first-line treatment (not including rituximab) compared with patients who reached only a PR has been reported in the recently published study with a longer follow-up (median 14.9 years).^10^ Also another recent study in follicular lymphoma patients treated with chemo-immunotherapy (R-CHVP-IFN) showed that the achievement of CR appears to be associated with an improved survival, although statistical significance was not reached.^13^ This issue by our opinion needs a further clarification on a larger group of patients having the same histological type of lymphoma.

We also confirmed some association between the earlier rituximab treatment and longer OS and DFS. These results are in agreement with the results of our previous study reporting outcomes of the treatment with various chemo-immunotherapy combinations.^14^ By that study it was confirmed that results of the treatment with rituximab and chemotherapy are in all aspects superior when the patients receive chemo-immunotherapy as the first-line treatment. The ORR was higher in the first-line treatment compared to the second or third/subsequent line treatment in patients with DLBCL, FL but not in MCL. The same was determined for DFS and OS which were longer in the first-line treatment compared to the second or third/subsequent line treatment in patients with DLBCL, FL and MCL. These observations seem logical at least in aggressive types of lymphomas where one can expect that previously untreated lymphoma patients would respond to treatment optimally as the possible mechanisms of resistance to treatment have not emerged yet.^15^ The impact of the primary treatment in indolent lymphoma patients on OS might be on the other hand interfered with the second or the third-line treatments which are very likely to be needed in the course of indolent lymphomas. But in general, various other studies have also reported better survival outcomes for the frontline treatment with rituximab both in indolent and in aggressive lymphomas which was simply confirmed with our research.^16^–^21^

In clinical praxis, there is no doubt about how to treat patients with CD20 positive aggressive lymphomas – they should receive rituximab in front-line therapy. On the contrary, the situation in indolent lymphomas is still somehow undefined since the recommendations for the treatment of indolent lymphomas in the last few years favour the first-line treatment with rituximab. Our results, however, demonstrate that in rituximab naïve patients also the second and the third-line treatment is justifiable.

In conclusion, our data indicate that a better response to the rituximab therapy presumably translates into an improved OS and DFS for patients with B-cell lymphomas. The early introduction of rituximab into the treatment (i.e. first-line treatment) might improve OS. The proposed first-line treatment with rituximab should be considered in both types of lymphomas – the aggressive and the indolent ones. Yet, in rituximab naïve patients with indolent lymphomas also the second or subsequent line of the treatment with rituximab should be taken in account.

## Figures and Tables

**TABLE 1.** Patients’ characteristics together with the distribution of patients according to the lines of treatment

**Patient’s characteristics**				
**Gender**	64 males (56.1%); 50 females (43.9%)			
**Age**	Median 58.5 years (range 19 to 82 years)			

**Histological type**	Number (%) of patients	Number of patients treated with rituximab as first-line treatment (%)	Number of patients treated with rituximab as second-line treatment (%)	Number of patients treated with rituximab as third or subsequent line of treatment (%)
**DLBCL**	42 (36.8)	33 (78.6)	8 (19)	1 (2.4)
**FL**	30 (26.3)	6 (20)	11 (37)	13 (43)
**CLL**	5 (4.4)	0	2 (40)	3 (60)
**MCL**	20 (17.5)	8 (40)	6 (30)	6 (30)
**MZL**	2 (1.8)	0	1 (50)	1 (50)
**Unclassified**	15 (13.2)	3 (20)	5 (33)	7 (47)

**Total**	114 (100)	50 (43.9)	33 (28.9)	31 (27.2)

DLBCL = diffuse large B-cell lymphoma, FL = follicular lymphoma, CLL = chronic lymphocytic leukemia, MCL = mantle cell lymphoma, MZL = marginal zone lymphoma, Unclassified = unclassified B-cell lymphoma

**TABLE 2.** The distribution of patients receiving rituximab according to the response quality

**Response quality**	**Number of patients**	**% of all patients**
**CR**	70	61.4
**PR**	17	14.9
**SD**	6	5.3
**PD**	19	16.7
**Unspecified**	2	1.8
**ORR**	87	76.3

**Total**	114	100

CR = complete response, PR = partial response, SD = stable disease, PD = progressive disease, ORR = overall response rate, Unspecified = response not specified

**TABLE 3.** The distribution of patients receiving rituximab according to the response quality and type of lymphoma

**Response quality**	**DLCBL**		**FL**		**MCL**		**Unclassified**	

	Number of patients	% of all patients	Number of patients	% of all patients	Number of patients	% of all patients	Number of patients	% of all patients
**CR**	29	69	22	73,3	9	45	8	53,3
**PR**	8	19	3	10	2	10	3	20
**SD**	0	0	1	3,3	4	20	1	6,7
**PD**	4	9,5	3	10	5	25	3	20
**Unspecified**	1	2,4	1	3,3	0	0	0	0
**ORR**	37	88,1	25	83,3	11	55	11	73,3

**Total**	42	36.8	30	26.3	20	17.5	15	13.2

*CR* = complete response, *PR* = partial response, *SD* = stable disease, *PD* = progressive disease, *ORR* = overall response rate, *Unspecified* = response not specified, *DLBCL* = diffuse large B-cell lymphoma, *FL* = follicular lymphoma, *MCL* = mantle cell lymphoma, *Unclassified* = unclassified B-cell lymphoma

**TABLE 4.** The distribution of patients receiving rituximab according to response quality and the line of treatment

**Response quality**	**All lines**		**1st line**		**2nd line**		**3rd line**	

	**Number of patients**	**% of all patients**	**Number of patients**	**% of all patients**	**Number of patients**	**% of all patients**	**Number of patients**	**% of all patients**
**CR**	70	61.4	36	72	21	63.6	13	41.9
**PR**	17	14.9	10	20	4	12.1	3	9.7
**SD**	6	5.3	1	2	2	6.1	3	9.7
**PD**	19	16.7	2	4	5	15.2	12	38.7
**Unspecified**	2	1.8	1	2	1	3	0	0
**ORR**	87	76.3	46	92	25	75.8	16	51.6

**Total**	114	100	50	43.9	33	28.9	31	27.2

*CR* = complete response, *PR* = partial response, *SD* = stable disease, *PD* = progressive disease, *ORR* = overall response rate
